# Supplementary material for: Patterns of Intron Gain and Loss in Fungi
Source: PLoS Biol. 2004 Nov 30;2(12):e422. doi: 10.1371/journal.pbio.0020422 (PMC532390; doi:10.1371/journal.pbio.0020422)
Supplement: Table S1 — Also available at http://genes.mit.edu/NielsenEtAl/. (4.3 MB ZIP). [file pbio.0020422.st001.zip › NielsenEtAl/html/777.html]

AN5839.1.NCU05802.1.MG04709.1.FG09875.1


```
 CLUSTAL W (1.82) Multiple Sequence Alignments - Introns Inserted


Sequence 1: NCU05802.1	1491 aa
Sequence 2: MG04709.1	1504 aa
Sequence 3: FG09875.1	1420 aa
Sequence 4: AN5839.1	1436 aa
Alignment Length: 1525 aa
Number Identitical Residues: 552 aa
Alignment Score (without introns) 30066


MG04709.1 	MAGGNNKKKKKPAGN-QRGFATTSIASKPQVRVIDPAEAQPSADGSKPGSAPPNAAPQAG
NCU05802.1	MAG--KKKSKKPAANPARGFATTSLPSKPREAAEDVAPPKPNSDTTKNN-------VQAS
FG09875.1 	MAGG-TKKKKKPAANPARGFATTSVASKPRPEVTEPE-SKPAVAKSQDAP------PSTQ
AN5839.1  	MPPN-SKKKKKPAANPARGFATVSVPSKVKP----TETSEPASTVDSKS-------VSES
          	*. . .**.****.*. *****.*:.** :        .:*     .          .  

MG04709.1 	GSADSSAGTQAPNGSGPQQQKQELTPEEFEKQLEESELQLLVEKHAAKVRRDAQRQRNRL
NCU05802.1	KDAPATNGTTAPTEAAKAAPEKTLSPEEFERQLEESELQLLVEKYAPKVRRDALRQKTRL
FG09875.1 	KDAPPSSSTAGNATAAANSQEAALSPEEFEKHLEESELQILVEKHATKTKKDALRQRNRL
AN5839.1  	DRPTPSEPTHTAPSSDKTPSLQNYTPEELERHLEDAELQILVEKYAAKCKSDAVRQASKL
          	  . .:  *     :         :***:*::**::***:****:*.* : ** ** .:*

MG04709.1 	ETDKRLLRGQAESLNTRKWLPPELMEQLLELIKGESRYAASGLASETSS--GKMPSEEDL
NCU05802.1	ETDRRLLRGQADSVNAKKWLPQDLMDHVLDLIQAESRFAASSLTSEGAT--SRLPPEEDL
FG09875.1 	DTDRRLFRSQADTINSFKWLPQDLMNHILDLIQAENRFTASSLSSSDSTNAGKMLPEEEM
AN5839.1  	ETERRVLRQQAVSVNHVEWLPSDVVSSILALAEAE----EQGLSPQPVRD-KRVVTEDEL
          	:*::*::* ** ::*  :*** :::. :* * :.*     ..*:..   .  :: .*:::

MG04709.1 	TIKLWTLQEALSGSGFPKDKVPALLQHILDIAPSIS-APKGDSIWGLDEALAWFARECDN
NCU05802.1	IIKLWTLQQTLEGADFPQEKIQPALQFALDIAPNISSTVKSDSVWGLDEVLDWLARECSK
FG09875.1 	IIRLWTLQQTLTAAEFPTERVAAVIRYILDIAPNVS-TTSRESIWGLEEALDWLARECRT
AN5839.1  	CMKLWVLKDTLLRLGFPENRVEEALKHVLIYFAGNFTVTNRDVVCNLEEALEWLAMHCSL
          	 ::**.*:::*    ** :::   ::. *   ..  :. . : : .*:*.* *:* .*  

MG04709.1 	DELPNYEKSAKMNGK-PLD1TPFDTP~LPSGATTPRLLEPDTKKKRKKDG----KSRATS
NCU05802.1	EELPDYSGRAKKS---QID1TPSETP~LPSGAVTPQLPELGPRGKKAKNNQTSRSSRAPS
FG09875.1 	DELPNYEPKSKPILKSTTD1TPQDSP~VPSRSSTPRHADPRNGRKNKSSGVKGRPVRNSP
AN5839.1  	DELPSYTQTNGQRKDADKN~VSWISD1RETSGKSAPKFAAGSKMQKAKPV-----VRENI
          	:***.*        .:  : ..  :    : . :.         :. .        *   

MG04709.1 	PRKVAVTCDEEIEPDELVPQYLDTRTKLYALQRPLLDAPKGKQKKLVVENGQSSSDELEI
NCU05802.1	PKRSKVVFDEYIEPDQLLPFYLETKTKLFEIQRPRLDTAKGKK-----GKPEANPSDPEE
FG09875.1 	DKKMAVACDSDIEPDELVSKYLESRAKLLEYERGRPEN----------LPINEHDEQLAI
AN5839.1  	TQPDPYDSDSSLDPDTLVPKYLDLQTRLYNLCPEIFDRPKKGKK----GQPAGATDDPQA
          	 :      *. ::** *:. **: :::*        : ..  ..           .:   

MG04709.1 	AKLQAKIAKIELDVLFEKDVATAQWREKKIILEREYSAAKKIKDEEAKAQAASQEPEPAV
NCU05802.1	ALLLAKIERVEKDILFDKYVAEQQWRDKKIDLEKDYAANKKKQVEEEQEEKK-QAQEETS
FG09875.1 	AKLEAKIRKIESDVLFDKYEADQQWKVQRVDLERQLAAAKHDRQDQAG-ENLVVEREPAS
AN5839.1  	LKLQRKISSIENDVLFDRKEAEYLWREKLDELRKEAAIFRRVDANAETEQVEKKESEPET
          	  *  **  :* *:**::  *   *: :   *.:: :  ::   :    :      *   

MG04709.1 	AQSAEDEIAREAEKMAAEILAEN-DNEEDALAGLFDSLPTNEVDPLTGKSSTVMTGADGV
NCU05802.1	TPTEVDDIAAEAERMAAELLAENPEDDDEALAGLFASLPVNEVDPTTGKTNTVMNNADGS
FG09875.1 	EKTDVGDISNEAERIAAEILAEV-NDEEDDIGGLFASLPQNEVDASTGESRTVVNSADGN
AN5839.1  	PPALD-------------------DEADDILGGMFQ------DEEPTLELGVITEELNKA
          	  :                     :: :: :.*:*        :  * :  .:    :  

MG04709.1 	KLTIHDFGKWTGISPSRALDEICRGR2DPGVKISYTAIPATAFAHQHLVHVLWSKAQEPF
NCU05802.1	RIQIRDFGKWTGVSPMRALEEACRSR~DNAVKISYHVLSDVSFASRHAVKILWSKPQEIP
FG09875.1 	QVVIRDFGKSTGVSPRRALEEACRSR2DSMAKITYTIVSDASFANRHSVDIIWTKPQDLP
AN5839.1  	SIATRDFGKWTGLSPRRVLEEACKAR2DSGCKVTYKDISTASHLNRMALEVRWSKPQEMP
          	 :  :**** **:** *.*:* *:.* *   *::*  :. .:.  :  :.: *:*.*:  

MG04709.1 	QLHDIPGVEVEMHPNQFVIKMKSIATPDSRQSEAYVATLAFFLVSGSSSKDEKAAMKLPP
NCU05802.1	APPDSDDIEVFTAPTQFVFKMVSIATPDAKQSEAYIATIALFLIFSSSAKEEKVAMRLPV
FG09875.1 	PPAASSEVEVVADHFRFTFTMTSIATPDSKQSESYISTAALFYTFSGNTKDEKVAIRLHH
AN5839.1  	SPFSHEAVTQKSTSYATFVSMDKIATPTSQQAEAYASTFALFILFPQNSKEGKAYMRLPA
          	       :          ..* .**** ::*:*:* :* *:*     .:*: *. ::*  

MG04709.1 	VWRDLWSELAEAKKSKADAADRE~ILKELRALVRKKQDQELEDGVIIQGAFRGRGARAAA
NCU05802.1	AWKDLWSEMAEARKSKADEADRA~VVKHLRDLVRKRMDQELEDGVIIQGAFKGRGQSRAQ
FG09875.1 	VWKDVWAELSEARKAQIDEKDRE0YGPIVR------------------------------
AN5839.1  	AWRDLWEEFANAKKVQDDEADKQ~VVKSLRKLIQENHGTFEDDVVLSDNFRKRNGTPRNR
          	.*:*:* *:::*:* : *  *:      :*    .  .   ..    ..     .     

MG04709.1 	TDSAENSDHDRNKAAAVGPEYYQRIWYDKSSSPRYQNMLRSRMQLPMWAFREQVLSAVDQ
NCU05802.1	TDSE--SDQERAKRNAQDPEYYQRIWLQKASTPRFQQMLASRMQLPMWQFRQQVVDTVKR
FG09875.1 	------------------SETYS--------------MQQSRMQLPMWHFREQVLEAVDG
AN5839.1  	SPVR-----AGAKEAPGINEQLSQIWMEKASTPAFQYMVQSRMQLPIWTFKNEILNTLNS
          	:           .  .   *  .    ..:::.  . *  ******:* *:::::.::. 

MG04709.1 	HQVVIVCGETGC~GKSTQVPSFLLEHQLAQGKACKIYCTEPRRISAISLARRVSEELGEG
NCU05802.1	EQVVIICGETGC~GKSTQVPSFLLEDQLMKGRNCKIYCTEPRRISALSLAKRVSEEIGEG
FG09875.1 	NQVVIVCGETGC2GKSTQVPSFLLEHQLSQGRPCKVYCTEPRRISAISLARRVSEELGEN
AN5839.1  	HRALIICSETGS~GKSTQIPSFILEHEMLSGRPCKIYVTEPRRISAISLARRVSEELGES
          	.:.:*:*.***. *****:***:**.:: .*: **:* ********:***:*****:**.

MG04709.1 	RNDLGTSRSLVGYSIRLEANTSRETRLVFA~TTGIVMRMLEGSNDLRDITHLVLDEVHER
NCU05802.1	RGDLGTPRSLVGYSIRLEANTSRETRLVYA~TTGIVMRMLEGSNDLNEITHLVLDEVHER
FG09875.1 	RNDLGTNRSLVGYSIRLEANTSRETRLVYA~TTGIVMRMLEGSNDLQEVTHLVLDEVHER
AN5839.1  	KNDVGTSRSLIGFAVRLESKVSPATRLVFA2TTGVVVRMLERPDDFQDITHVVLDEVHER
          	:.*:** ***:*:::***::.*  ****:* ***:*:**** .:*:.::**:********

MG04709.1 	SIDSDFLLIVLKKLMTRRKDLKVVLMSATVDADRFSNYLDGAPVLNVPGRTFPVQVNFLE
NCU05802.1	SIDSDFLLIVLKKLLARRKDLKVVLMSATVDAERFSKYLGGAPVLSVPGRTFPVKVAYLE
FG09875.1 	TIDSDFLLIVLKKLLVRRKDLKVVLMSATVDADRFSAYLGGAPVLNVPGRTFPVQVRYLE
AN5839.1  	SIDSDFLLIVLRRLMQKRQDLKLILMSATVDANRFSTYLGGVPVLNIPGRTFPVETKFLE
          	:**********::*: :*:***::********:*** **.*.***.:*******:. :**

MG04709.1 	DAVELTGYTIDQKVPQE-RLVEIDEDAEVEPESSSKTELLKSLKGYSTRTRNTLAQMDEY
NCU05802.1	DAVELTGYTLDQRNPVASKLTELDDEADAEVDTSSKPELIQSLRNYSARTRNTLAQMDEY
FG09875.1 	DAIELTGYAPADSEPDK--MLDLDDDPAESEGENTKSDISKSLASYSSRTKSTLTQIDEY
AN5839.1  	DAIELTQYRTTENESNV---ADDEYEDDAETSQGETTGVAATLENYSKQTRETIMNFDEY
          	**:*** *   :  .      : : :   .   . .. :  :* .** :*:.*: ::***

MG04709.1 	KIDFDLIVQLISRIASDPNYQDYSKAILVFLPGIAEIRTLNDLLLGDRSFQQNWLVYPLH
NCU05802.1	QIDFDLIVQLISTIATHPDYVDFSKAILVFLPGIAEIRTLNDMLLGDKAFADHWLVYPLH
FG09875.1 	RIEFDLILQLIAQIAINDQLQDFSKAILVFLPGIAEIRTLNDMLLGDPRFAKDWLVYPLH
AN5839.1  	RLDYQLIKKLLMKLATAPEMEYYSKAILVFLPGMAEIRRLNDELLSEPTFQHGWIIHALH
          	:::::** :*:  :*   :   :**********:**** *** **.:  * . *:::.**

MG04709.1 	STIATEEQEAAFLVPPHGMRKIVLATNIAETGITIPDVTCVIDTGKHREMR~FDERRQMS
NCU05802.1	STIATEDQEAAFLVPPPGLRKIVLATNIAETGITIPDVTCVIDTGKHREMR~FDERRQLS
FG09875.1 	STIATEDQESAFLVPPPGIRKIVLATNIAETGITIPDVTCVIDTGKHREMR~FDERRQLS
AN5839.1  	SSIASDEQEKAFVVPPDGMRKIVIATNIAETGITIPDITAVIDAGKEKTMR2FDERRQLS
          	*:**:::** **:*** *:****:*************:*.***:**.: ** ******:*

MG04709.1 	RLIDTFISRANAKQRRGRAGRVQQGLCFHMFTKYRHDAI~MADQQTPEMLRLSLQDLAIR
NCU05802.1	RLIDTFISRANAKQRRGRAGRVQEGLCFHMFTKHRHDNI~MSDQQTPEMLRLSLQDLAIR
FG09875.1 	RLIDTFISRANAKQRRGRAGRVQEGLCFHMFTKFRHDQL~MSDQQTPEMLRLSLQDLAIR
AN5839.1  	RLVEAFISRANAKQRRGRAGRVQRGICFHMFTKHRHDNL0LAEQQTPEMLRLSLQDLVLR
          	**:::******************.*:*******.*** : :::**************.:*

MG04709.1 	VKICKIGGIEETLSKALDPPSAKNIRRAIDALIDVRALTQG-EDLTPLGNQLARLPLDVF
NCU05802.1	VKICKIGGIEETLGEALDPPSAKNIRRAIDALVDVRALTASSEELTPLGIQLARLPLDVF
FG09875.1 	VKICKIGGIEETLGDALDPPSAKNIRRAVDALVDVRALTQT-EELTPLGYQLARLPLDVF
AN5839.1  	VKICKLGEVEPTLLEALDPPSSKNIRRAIDALKEVKALTST-ENLTPLGQQLAKLPLDVF
          	*****:* :* ** .******:******:*** :*:***   *:***** ***:******

MG04709.1 	LGKLILLGSIFKCLDMAITVAAILSSKSPFVAPFGQRSQADTVRLGFRRG~DSDILTVYN
NCU05802.1	LGKLILLGAIFKCLDMAITVAAILSSKSPFVAPFGQRQQANTVRMGFRKG~DSDLLTVYN
FG09875.1 	LGKLILYGVIFKCLDMAITTAAILSSKSPFSAPFGQRTQADNARMAFRRG~DSDLLTIYN
AN5839.1  	LGKLIIHGAFFKCLDAAISIAAILSCKSPFVNTMGSNAQKEAVRLGFRRG1DSDLLTVYN
          	*****: * :***** **: *****.****  .:*.. * : .*:.**:* ***:**:**

MG04709.1 	AYLSWKRVCLAATGNSSQEFQFCRKNFLSQQTLANIEDLKGQLLVSLVDSKFLSMTEDER
NCU05802.1	AYQSWKRVCQSSTSGG-AEFQYCRKNFLSPQTLANIEDLKGQLLVSVADSGFLQLTTEER
FG09875.1 	AYLAWKRVCQSAGGGG-KEFQFCRKNFLSQQTLANIEDLKGQLLTSLADSGFLSLTEEER
AN5839.1  	AYCAWKRTRSTP---GANEYAFCRKNFLSPQTLLNIEDVKLQLMVSIADAGLVTLDTNQK
          	** :***.  :.   .: *: :******* *** ****:* **:.*:.*: :: :  :::

MG04709.1 	KNLSR~LRYASGGNRRRKQVFFEMPQRVDVNSENDLVSQAVLSWSFYPKLLVRDVAGGKG
NCU05802.1	QVLNR~LRFGG---KRRYQAFYEVPQRVNINSDNELIAQSVIAWSFYPKLLVRDVPGSKG
FG09875.1 	RALSR~ARFSGG-RGRRQQQFYDIPRRVNLNSDNDVVSASVIAWSFYPKILVRDAPGSKG
AN5839.1  	ASLNR2ARSTR------QRHFFTTPPSHDTNNANDTLIQSVIAWSFYPKLLTREG---KG
          	  *.*  *          : *:  *   : *. *: :  :*::******:*.*:    **

MG04709.1 	LRNVGNNQNISLHRSSVN--------RGHNEIRWMSYYHMMQSTK~S2ALNAHETTAVEP
NCU05802.1	LRNVGNNQNISLHPSSVN--------KGHNELRWLSYYNIMQTKG2S~VYNAHETTAVDP
FG09875.1 	LRNIGTNQSISLHPSSVN--------RGRLDLRWLSYYHIMQSR-~A2VYHAHEATAVEA
AN5839.1  	WRNIANNQSVTLHPTSVNRVPSASSERSPGQSQYLSYYHIMQGRN~R~KYNAFETSAVED
          	 **:..**.::** :***  .::::.:.  : :::***::**        :*.*::**: 

MG04709.1 	FAIALLCGDVRCD0MYSGVFVMDGNRARFAAPDWKTVLVVKTLRSRLRELLTRSFKNPGK
NCU05802.1	FAVALLCGDVRAD~MYSGVLVLDGNRARFAVSDWKTMLVVKMLRTRLKEVMARSFKNPGK
FG09875.1 	FPIALLCG----D0MYSGVIILDGNRGRFVVPDWKTMLVIKVLRTRLRELLTRSFKQPGK
AN5839.1  	WAVAVLCGEGDFK0MYPGVLSIDTNRLRFSLRDWKSMLAMKVLATRIREILANVFRDPQQ
          	:.:*:***.   . **.**: :* ** **   ***::*.:* * :*::*:::. *::* :

MG04709.1 	LPTPQHQHWLEAWQQIYEQAAAKDAAKAAAAAAAGGS-
NCU05802.1	LPTAQHERWLEVWQRIFTMAMEQREKAAATASTVSVAA
FG09875.1 	LPTAQQEKWLDVWQRIFTQDFGQDRSTTGMTIKA----
AN5839.1  	PLSYKQRQWMDIWQGIWQARM-----------------
          	  : ::.:*:: ** *:
```
